# Association between sleep duration and co-existing chronic conditions in adults: insights from the 2017–2018 Canadian Community Health Survey

**DOI:** 10.3389/frsle.2026.1714511

**Published:** 2026-09-09

**Authors:** Shirmin Bintay Kader, Jubayer Mumin, Nahin Shakurun, Sumsun Nahar Zinia, Zoe Schipper, Nusrat Sharmin Ritu, Sheikh Ahmedul Haque, Md. Marufur Rahman

**Affiliations:** 1 Department of Community Health and Epidemiology, University of Saskatchewan, Saskatoon, SK, Canada; 2 Department of Global Public Health, Karolinska Institute, Stockholm, Sweden; 3 Directorate General of Health Services (DGHS), Ministry of Health and Family Welfare, Dhaka, Bangladesh; 4 NISPOM RT-PCR Lab, National Institute of Preventive and Social Medicine (NIPSOM), Dhaka, Bangladesh; 5 Research Division, Platform Doctors Foundation, Dhaka, Bangladesh; 6 Division of Clinical Medicine, School of Medicine and Population Health, The University of Sheffield, Sheffield, United Kingdom

**Keywords:** Canadian Community Health Survey (CCHS), chronic disease—epidemiology, multimobility, sleep duration, sleep health

## Abstract

**Background:**

Sleep duration plays a crucial role in overall health, with far-reaching implications for individuals globally. Although research has extensively explored the association between sleep and chronic conditions, few studies have investigated the association between multiple co-existing chronic conditions on sleep duration. This study examines the association between sleep duration and the prevalence of chronic conditions in the Canadian adults from four provinces and two territories.

**Methodology:**

Canadian Community Health Survey (CCHS) 2017–2018, a population-based cross-sectional survey, was used for this study. The outcome variable, sleep duration, was assessed based on the question “Number of hours per night usually spent sleeping?” and categorized into three groups: “In between 7 and 9 h,” “Short sleep duration (< 7 h),” and “Long sleep duration (>9 h).” The primary predictor, chronic conditions, was aggregated with a response of “yes” and subsequently categorized into no chronic condition, one chronic condition, two chronic conditions, and three or more chronic conditions, based on 12 recorded chronic conditions.

**Results:**

Approximately 41% of respondents reported short sleep duration, while 7.73% reported long sleep duration. Compared with respondents with fewer or no chronic conditions, those with three or more co-existing chronic conditions had significantly higher odds of reporting abnormal sleep duration. Specifically, respondents with three or more chronic conditions were 1.70 times more likely to report short sleep duration (95% CI: 1.52–1.91) and 2.58 times more likely to report long sleep duration (95% CI: 1.96–3.41). The odds of both short and long sleep duration increased progressively with a higher number of chronic conditions, indicating a graded association between chronic conditions and abnormal sleep duration.

**Conclusion:**

The study underscores a strong association between the number of chronic conditions and sleep duration among adults, highlighting that multimorbidity is associated with abnormal sleep patterns.

## 1 Introduction

Sleep has a substantial effect on people's health. Sleep disorders such as abnormal sleep duration, sleep quality, and insomnia are affecting people's health worldwide (Benbir et al., 2015; Ford et al., 2015; Senaratna et al., 2016; Sheehan et al., 2019). According to the American Academy of Sleep Medicine and Sleep Research Society, between 7 and 9 h of sleep is recommended for adults (Watson et al., 2015), and this is noted to decline with age among Canadians (Chang et al., 2018). Short sleep durations are increasing among Canadian adults, which is alarming (Chaput et al., 2009). Sleep disorders are strongly linked to chronic illnesses through multiple biological, physiological, and behavioral pathways (Deurveilher and Semba, 2019; Butris et al., 2023; Banks and Dinges, 2007). Studies have demonstrated that sleep duration is associated with various health outcomes, including cardiovascular and respiratory conditions such as stroke (Khot and Morgenstern, 2019; McDermott et al., 2018), heart disease (Nelson and Trupp, 2015), and asthma (Prasad et al., 2020), as well as neurological diseases, including dementia (Wennberg et al., 2017). Additionally, researchers found a substantial association between sleep duration and deteriorating outcomes from hypertension (HTN) (Gottlieb et al., 2006; Yang et al., 2021), obesity (Seo and Shim, 2019), and diabetes mellitus (DM) (Chaput et al., 2009; Wang et al., 2020). A population-based study by Sivertsen et al. (2014) among Norwegian adults showed that short sleep duration is significantly associated with arrhythmia, myocardial infarction, asthma, osteoporosis, and depression.

Similarly, people who suffered from chronic illnesses, such as cardiovascular disease (CVD), reported higher odds of having insomnia (Ali et al., 2023). Many chronic conditions are associated with pain, inflammation, hormonal dysregulation, autonomic nervous system activation, and medication side effects, all of which can disrupt normal sleep patterns (Haack et al., 2020). Additionally, the psychological distress, functional limitations, and reduced physical activity resulted due to chronic illnesses may further compromise sleep duration and quality (Dubinina et al., 2021; Bulthuis et al., 2004). As a result, sleep disturbances are common among individuals with chronic diseases and may, in turn, exacerbate disease progression and adverse health outcomes.

Various sociodemographic and biological factors, including age group (Ancoli-Israel, 2009), gender (Cappuccio et al., 2007), marital status (Stranges et al., 2008), education (Yoon et al., 2015), employment (Yoon et al., 2015), physical activity (Ali et al., 2023), and Body Mass Index (BMI) (Yoon et al., 2015), are correlated with sleep duration. A retrospective longitudinal study found that respondents with either short (< 6 h) or long (>8 h) sleep durations were twice as likely to have more than two chronic conditions among Canadians aged 45–54 (Nicholson et al., 2020). Systematic reviews have demonstrated that both short and long sleep durations are significantly associated with multiple chronic conditions, such as coronary heart disease, diabetes mellitus and hypertension (Itani et al., 2017; Jike et al., 2018). Short sleep duration is associated with chronic conditions in individuals under 65 years of age (Itani et al., 2017), whereas long sleep duration is linked with coronary heart disease in adults over 65 years (Jike et al., 2018).

A recent systematic review of 17 studies (Zhou et al., 2023) indicated that both short sleep duration (OR = 1.49) and long sleep durations (OR = 1.21) are associated with multimorbidity, defined as the coexistence of two or more chronic conditions at the same point in time (Johnston et al., 2019). While numerous studies have established the relationship between sleep duration and specific chronic conditions (Chaput et al., 2009; Khot and Morgenstern, 2019; McDermott et al., 2018; Nelson and Trupp, 2015; Gottlieb et al., 2006; Yang et al., 2021; Seo and Shim, 2019; Wang et al., 2020; Itani et al., 2017; Jike et al., 2018), the current body of knowledge has yet to investigate the association between multiple concurrent chronic conditions and sleep duration among Canadians. Research shows that Canadians exhibit a high prevalence of multimorbidity, ranging from 24% to 31%, which may also significantly influence sleep duration (Nicholson et al., 2020). Therefore, this study aims to examine the association between the number of co-existing chronic conditions (multimorbidity) and sleep duration among Canadian adults (from a subpopulation from four provinces and two territories), with sleep duration as the primary outcome.

## 2 Methods

### 2.1 Study design, sample, and participants

For this study, we used publicly available microdata files from the Canadian Community Health Survey (CCHS): 2017–2018. CCHS is a complex cross-sectional survey that collects data from all 10 of Canada's provinces and three territories. As the CCHS is cross-sectional in design, this study aimed to assess associations between chronic conditions and sleep duration and does not allow for causal inference or determination of temporal relationships. It collected information from a nationally representative sample of individuals aged 12 years and older, including sociodemographic status, health status, and sleep-related information. A subsample was collected from selected provinces in Prince Edward Island, Quebec, Alberta, and British Columbia, as well as territories in Yukon and Nunavut. Detailed details regarding the sampling method, design, data collection techniques, and procedures can be found in the original source (Statistics C, 2020).

The present study used the same CCHS cycle as a previous publication from our research group (Kader et al., 2024, 2025). To ensure transparency regarding potential overlap, we note that current and previous studies included some overlapping participants; however, the objectives, analytical approaches, and interpretation of findings were distinct. Our previous studies examined the relationship between sleep and self-rated health (SRH) from two complementary perspectives. The first study investigated the association between sleep duration and SRH, with multimorbidity examined as a potential mediator of this association (Kader et al., 2024). The second study assessed the joint effect of sleep duration and sleep quality on SRH (Kader et al., 2025). In contrast, the current study shifts the focus from SRH to chronic disease burden, specifically investigating the association between the number of chronic conditions and sleep duration.

A total of 113,290 respondents participated in the study. To examine the potential association between sleep duration and chronic conditions, we used data from a subsample of 56,675 individuals with available sleep data. The analytic sample was restricted to adult respondents (aged ≥ 18 years) who provided complete information on sleep duration and chronic conditions. As sleep-related questions were administered to a subset of CCHS respondents, approximately 50% of the original survey sample was eligible for inclusion in the present analysis, which ended up with a final analytical sample consisting of 52,378 participants.

### 2.2 Ethics approval and consent to participate

This study used publicly available, de-identified microdata from the CCHS. The CCHS data collection protocol received initial ethical approval from Statistics Canada's Research Ethics Board. As the present analysis involved secondary analysis of publicly available, anonymized data, additional institutional research ethics board approval was not required.

### 2.3 Study measures

Our outcome variable was sleeping duration. Participants were asked to respond to this open-ended question: “Number of hours per night usually spent sleeping.” This measure captures participants' typical nightly sleep duration and does not distinguish between weekdays and weekends, nor does it specify a specific recall period. Subsequently, the responses were re-coded into three categories: “Between 7 and 9 h,” “Short sleep duration (< 7 h),” and “Long sleep duration (>9 h).” The primary predictor variable was the number of existing chronic conditions, determined by responses indicating “yes” to any chronic condition under the chronic condition inclusion flag in the CCHS 2017–2018 data. Initially, we considered 13 conditions (Asthma, COPD, Arthritis, Hypertension, Hyperlipidemia, Heart Disease, Stroke, Diabetes, Cancer, Allergies, Mood Disorder, Anxiety, and Hay Fever), as CCHS has collected the information on the original survey. However, because no valid responses were recorded for hay fever (zero response). As a result, multimorbidity was assessed using the remaining 12 chronic conditions. These chronic conditions were reported on previously published research (Kader et al., 2024; Geda et al., 2021; Thomander et al., 2022). Furthermore, each year during the survey, CCHS considers the common chronic conditions among the Canadian population. Public Health Agency of Canada reports that 44% of Canadian adults aged 20 years and older have at least one of the 10 most common chronic conditions, all of which were included in CCHS (Government of Canada, 2019). Frequencies of the chronic conditions considered in our study are listed in the Supplementary Figure 1. A cumulative “yes” score was calculated to determine the number of chronic diseases diagnosed among respondents. Later, the cumulative score was categorized into four groups: *no chronic condition*, one *chronic condition*, two *chronic conditions*, and three *or more chronic conditions*. Based on the literature review, we considered the following covariates: sociodemographic factors (age, sex, ethnicity, immigration status, marital status, and highest level of education), socioeconomic position (employment status and annual income), and behavioral risk factor (BMI and physical activity levels). Physical activity was categorized according to the Canadian Physical Activity Guidelines (CPAG; Government of Canada, 2025). Respondents were classified as having no physical activity, below the recommended level, or meeting/exceeding the recommended level of physical activity. In the Results section, the term “above recommendation” refers to participants who met or exceeded the recommended level of physical activity. A comprehensive list of variables is available on the STAT-Canada website (Statistics C, 2020).

### 2.4 Statistical analysis

For statistical analysis, we used Stata version 15 (StataCorp, 2017), and for graphical presentation, we used RStudio (Posit, 2024). Survey weight was considered for all analyses. The principle underlying the use of survey data weights was to preserve the representativeness of the sample's characteristics. According to CCHS data, each respondent in the sample represents 50 individuals in the population. For this dataset, sampling weight was cumulated by Statistics Canada and presented as a variable (WTS_M) in the microdata file. Population distribution, frequency of chronic diseases, and distribution of sample characteristics across sleep duration and chronic conditions were presented as weighted percentages. To identify the sociodemographic, socioeconomic, and behavioral risk factors associated with sleep duration and chronic conditions, we conducted a chi-square test. A multinomial logistic regression, both crude and adjusted for sociodemographic, socioeconomic, and behavioral risk factors, was conducted to examine the association between the number of chronic conditions (primary exposure) and sleep duration (outcome), with sleep duration of 7–9 h serving as the reference category. The final adjusted model included age, sex, ethnicity, marital status, education, income, employment status, immigrant status, body mass index (BMI), physical activity, smoking status, and alcohol consumption. Potential confounders were selected based on their established associations with multimorbidity and sleep health outcomes reported in previous literature. Additionally, we assessed the *p*-value for trend across the ordinal categories of multimorbidity to evaluate whether there was a progressive increase in the association across increasing levels of multimorbidity. To assess the linear trend, the multimorbidity variable (0, 1, 2, or ≥3 chronic conditions) was entered into the multinomial logistic regression model as an ordinal variable coded 0–3, and the corresponding Wald test *p*-value was reported as the *p*-value for trend.

## 3 Results

### 3.1 Sample demographics

The sample was predominantly composed of middle-aged adults, with 41.97% aged 40–64 years, while younger (18–19 years) and older (≥75 years) adults represented smaller proportions (Supplementary Table 2). Participants were nearly equally distributed by sex (49.62% male, 50.39% female), and most were White (79.39%), employed (77.13%), had an annual household income >$60,000 (52.12%), and held a post-secondary degree (64.50%) Nearly two-thirds of the participants (64.50%) held a post-secondary degree or were married (62.96%) at the time of data collection. Approximately one-fourth of the participants were immigrants (24.30%) or obese (25.73%). Nearly 60% of the respondents (57.54%) reported physical exercise above the recommended level (Table 1). Overall, 51% reported no chronic conditions, whereas 23% reported one, 14% reported two, and 12% reported more than three. Short sleep duration was reported by 40.96% of participants, whereas 7.73% reported long sleep duration (Table 1).

**Table 1:** Weighted distribution (weight %) of participants' socio-demographic characteristics: CCHS 2017–18, Canada.

Variables	Weighted % (95% CI)
Age group	
< 40 years	37.27 (36.57–37.96)
40–64 years	41.97 (41.28–42.66)
65 and above	20.77 (20.3–21.24)
Sex	
Male	49.61 (48.91–50.3)
Female	50.39 (49.7–51.09)
Ethnicity	
White	79.39 (78.72–80.04)
Non-White	20.61 (19.96–21.28)
Immigration status	
Non-immigrant	75.70 (75.04–76.35)
Immigrant	24.3 (23.65–24.96)
Marital status	
Married/common law	62.96 (6.23–63.63)
Widowed/divorced/separated	12.63 (12.27–13)
Single	24.41 (23.79–25.03)
Highest education	
Less than secondary school	12.08 (11.66–12.51)
Secondary school graduate	23.43 (22.82–24.04)
Post-secondary degree	64.50 (63.82–65.16)
Income (annual household)	
No income	6.78 (6.47–7.11)
Less than $20,000	13.48 (13.07–13.91)
$20,000–$40,000	14.39 (13.93–14.86)
$40,000–$60,000	13.23 (12.77–13.69)
More than $60, 000	52.12 (51.43–52.81)
Employment (working status-last 12 month)	
Yes	77.13 (76.56–77.7)
No	22.87 (22.3–23.44)
Body mass index (BMI)	
Normal/underweight	38.56 (37.87–39.26)
Overweight	35.71 (35.03–36.4)
Obese	25.73 (25.11–26.35)
Physical activity	
No activity	19.33 (18.81–19.87)
Below recommendation	23.13 (22.54–23.72)
Above recommendation	57.54 (56.85–58.23)
No. of chronic conditions	
No chronic condition	51.12 (50.43–51.81)
One chronic condition	23.48 (22.90–24.06)
Two chronic conditions	13.75 (13.30–14.21)
Three or more chronic conditions	11.65 (11.28–12.04)
Sleep duration	
Less than 7 h	40.96 (40.28–41.65)
In between 7 and 9 h	51.31 (50.61–52.01)
More than 9 h	7.73 (7.36–8.11)

### 3.2 Prevalence of sleep duration across sample characteristics

Table 2 shows the weighted distribution of the participants' sociodemographic, socioeconomic position, and behavioral factors by sleep duration. The prevalence of short sleep duration was highest (43.66%) among participants aged 40–64 years. By contrast, the highest prevalence of long sleep duration (15.97%) was observed among participants aged>65 years. Sleep duration patterns were similar by sex (Table 2). Non-white (47.90%) and immigrant (44.88%) respondents reported a higher prevalence of short sleep duration than their counterparts, white (38.93%) and non-immigrant (39.71%) respondents. On the other hand, the prevalence of long sleep duration was similar across ethnicity and immigration status groups.

**Table 2:** Distribution of participants' socio-demographic characteristics (in terms of weighted percentages) by sleeping hours: CCHS 2017–18, Canada.

Variables	Less than 7 h % (95% CI)	In between 7 and 9 h % (95% CI)	More than 9 h % (95% CI)
Age group			
< 40 years	41.07 (39.87–42.29)	53.13 (51.91–54.36)	5.79 (5.25–6.38)
40–64 years	43.66 (42.57–44.76)	50.98 (49.87–52.09)	5.36 (4.84–5.93)
65 and above	35.32 (34.21–36.45)	48.7 (47.53–49.88)	15.97 (15.03–16.96)
Sex			
Male	42.29 (41.3–43.29)	50.56 (49.56–51.57)	7.14 (6.67–7.65)
Female	39.66 (38.72–40.6)	52.04 (51.08–53.01)	8.30 (7.75–8.88)
Ethnicity			
White	38.93 (38.19–39.67)	53.17 (52.41–53.92)	7.90 (7.51–8.32)
Non-White	47.90 (46.03–49.77)	45.28 (43.42–47.14)	6.83 (5.88–7.91)
Immigration status			
Non-immigrant	39.71 (38.96–40.46)	52.53 (51.77–53.3)	7.76 (7.36–8.17)
Immigrant	44.88 (43.28–46.49)	47.64 (46.03–49.25)	7.48 (6.65–8.42)
Marital status			
Married/common law	40.71 (39.83–41.59)	52.55 (51.65–53.44)	6.74 (6.31–7.21)
Windowed/divorced/separated	42.99 (41.52–44.46)	46 (44.53–47.49)	11.01 (10.02–12.09)
Single	40.54 (39.07–42.01)	50.93 (49.43–52.42)	8.54 (7.72–9.43)
Highest education			
Less than secondary school	41.34 (39.53–43.18)	42.15 (40.37–43.96)	16.51 (15.13–17.98)
Secondary school graduate	42.12 (40.64–43.61)	49.56 (48.05–51.06)	8.33 (7.52–9.21)
Post-secondary degree	40.64 (39.78–41.5)	53.75 (52.88–54.62)	5.61 (5.22–6.03)
Income			
No income	43.25 (40.9–45.62)	46.55 (44.15–48.96)	10.21 (8.97–11.59)
Less than $20,000	41.46 (39.86–43.07)	48.17 (46.53–49.83)	10.37 (9.44–11.38)
$20,000–$40,000	40.27 (38.58–41.99)	50.24 (48.49–51.98)	9.49 (8.51–10.58)
$40,000–$60,000	39.73 (37.94–41.56)	52.28 (50.42–54.13)	7.99 (7.05–9.04)
More than $60, 000	41.02 (40.01–42.04)	52.82 (51.79–53.84)	6.16 (5.66–6.71)
Employment (working status-last 12 month)			
Yes	42.81 (41.95–43.67)	52.83 (51.96–53.69)	4.36 (4–4.76)
No	38.06 (36.72–39.42)	48.69 (47.33–50.07)	13.24 (12.27–14.28)
BMI			
Normal/underweight	38.27 (37.17–39.39)	56.56 (55.42–57.69)	5.17 (4.7–5.69)
Overweight	43.03 (41.82–44.23)	51.98 (50.77–53.18)	5 (4.54–5.5)
Obese	42.17 (41.47–42.88)	47.81 (46.43–49.19)	5.35 (4.74–6.02)
Physical activity			
No activity	38.6 (37.16–40.07)	47.3 (45.8–48.81)	14.09 (13.06–15.2)
Below recommendation	40.4 (38.99–41.83)	52.5 (51.05–53.94)	7.10 (6.38–7.91)
Above recommendation	42.07 (41.14–43.01)	52.41 (51.46–53.35)	5.52 (5.09–5.98)

The percentages for married (40.71%), widowed (42.99%), and single respondents (40.54%) in the short sleep duration group were nearly identical. The percentage of married respondents who slept for a long duration was only 6.74%, whereas this was 11.01% for widowed respondents (Table 2). Similarly, in terms of educational attainment, the percentage of respondents from the short sleep duration group was similar across the groups; however, long sleep duration was more common in those with a secondary school degree (16.51%) than with a post-secondary degree (5.61%), and it was statistically significant (*p* < 0.001; Table 2).

Considering socioeconomic factors, Table 2 shows a decreasing trend in the prevalence of long sleep duration (Table 2). With increasing annual household income, the prevalence of sleeping hours more than 9 h decreased from 10.21% to 6.16%. About 38.06% of unemployed respondents reported short sleep, compared to 42.81% of employed respondents. On the other hand, 13.24% of respondents who were unemployed said they had long sleep duration, compared to 4.36% of respondents who were employed, which was significantly difference (*p* < 0.001) across the group (Table 2). Respondents who were overweight reported the highest prevalence (43.03%) of short sleep duration, followed by obese (38.27%) respondents, whereas long sleep duration was similar across BMI categories (Table 2). Long sleep was more prevalent among those reporting no physical activity compared with those exceeding recommended activity levels (14.09% vs. 5.52%, *p* < 0.001). Overall, sleep duration differed significantly across sociodemographic, socioeconomic, and behavioral characteristics (*p* < 0.001).

### 3.3 Prevalence of chronic conditions across sample characteristics

Table 3 presents the weighted distribution of the participant's sociodemographic, socioeconomic position, and behavioral factors by the number of chronic conditions. The prevalence of number of chronic conditions increased with age. Nearly one-third (29.34%) of respondents aged 65 years or above reported having three or more chronic conditions, whereas only 2.26% of respondents aged less than 40 years had three or more chronic conditions. Females presented a slightly higher prevalence (Female: Male 12.81% vs. 10.48%) of three or more chronic conditions than males. Among racial groups, 12.84% of White respondents reported having three or more chronic conditions, compared with 6.15% of non-White respondents. Similarly, 12.55% of non-immigrants reported three or more chronic conditions, whereas the prevalence was lower among immigrants (8.87%). The highest prevalence (24.20%) of three or more chronic conditions was reported among the widowed or separated groups. The respondents struggling by socio-economical context reported the highest prevalence for three or more chronic conditions (less than secondary education (23.70%), no income (20.01%), and unemployed (22.48%). Participants who were obese reported nearly three times higher prevalence (19.19%) of three or more chronic conditions than normal/underweight respondents (6.45%). Respondents who reported no physical activity had the highest prevalence of three or more chronic conditions (21.24%) compared with those who reported doing physical activity above the recommended level. In contrast, only 8% of respondents who engaged in physical activity above the recommended level reported three or more chronic conditions.

**Table 3:** Weighted distribution (weight %) of participants' socio-demographic characteristics by chronic conditions: CCHS 2017−18, Canada.

Variables	No chronic condition weight % (95% CI)	One chronic condition weight % (95% CI)	Two chronic condition weight % (95% CI)	Three or more chronic conditions weight % (95% CI)
Age group^*^				
< 40 years	73.86 (72.79–74.89)	17.37 (16.48–18.3)	6.52 (5.97–7.11)	2.26 (1.94–2.62)
40–64 years	47.33 (46.22–48.44)	26.92 (25.95–27.91)	14.51 (13.76–15.29)	11.24 (10.62–11.89)
65 and above	17.98 (17.09–18.92)	27.48 (26.44–28.54)	25.19 (24.14–26.27)	29.34 (28.32–30.39)
Sex^*^				
Male	53.38 (52.38–54.37)	23.37 (22.55–24.22)	12.77 (12.15–13.42)	10.48 (9.97–11.01)
Female	48.9 (47.9–49.86)	23.58 (22.78–24.39)	14.71 (14.07–15.38)	12.81 (12.2–13.39)
Ethnicity^*^				
White	47.48 (46.72–48.23)	24.78 (24.14–25.43)	14.91 (14.4–15.44)	12.84 (12.4–13.29)
Non-White	66.27 (64.5–68)	18.37 (16.97–19.85)	9.21 (8.18–10.35)	6.15 (5.4–6.99)
Marital status^*^				
Married/common-law	50.63 (49.74–51.52)	24.79 (24.04–25.56)	13.69 (13.1–14.29)	10.9 (10.41–11.4)
Widowed/divorced/separated	30.51 (29.1–31.96)	24.96 (23.73–26.24)	20.32 (19.11–21.59)	24.2 (23.05–25.4)
Single	63.07 (61.66–64.46)	19.36 (18.22–20.54)	10.47 (9.68–11.33)	7.1 (6.48–7.78)
Highest education^*^				
Less than secondary school	32.02 (30.26–33.84)	25.06 (23.44–26.76)	19.55 (18.16–21.02)	23.37 (22–24.78)
Secondary school graduate	50.05 (48.55–51.56)	23.16 (21.97–24.39)	14.77 (13.76–15.84)	12.02 (11.22–12.86)
Post-secondary degree	55.47 (54.61–56.32)	23.22 (22.5–23.95)	12.19 (11.66–12.73)	9.13 (8.7–9.57)
Income^*^				
No income	44.16 (41.71–46.65)	21.28 (19.39–23.3)	14.55 (13.25–15.96)	20.01 (18.5–21.6)
Less than $20,000	40.31 (38.64–41.99)	22.98 (21.66–24.36)	16.98 (15.88–18.14)	19.73 (18.55–20.97)
$20,000–$40,000	46.36 (44.6–48.12)	23.41 (22.02–24.86)	15.64 (14.48–16.87)	14.59 (13.55–15.7)
$40,000–$60,000	51.2 (49.35–53.05)	23.3 (21.85–24.81)	13.78 (12.61–15.03)	11.73 (10.73–12.81)
More than $60, 000	56.11 (55.1–57.12)	23.96 (23.1–24.84)	12.28 (11.62–12.96)	7.65 (7.18–8.15)
Employment (working status-last 12 month)^*^				
Yes	60.86 (60.02–61.69)	22.72 (22.02–23.44)	10.54 (10.03–11.07)	5.88 (5.53–6.26)
No	32.53 (31.21–33.88)	25.38 (24.17–26.61)	19.61 (18.59–20.68)	22.48 (21.43–23.56)
Immigration status^*^				
Non-immigrant	48.29 (47.52–49.05)	24.44 (23.8–25.09)	14.72 (14.21–15.24)	12.55 (12.12–13)
Immigrant	59.49 (57.92–61.04)	20.64 (19.36–21.98)	11 (10.05–12.03)	8.87 (8.12–9.67)
Body mass index (BMI)^*^				
Normal/underweight	61.59 (60.5–62.67)	22.22 (21.3–23.17)	9.74 (9.13–10.38)	6.45 (6.01–6.92)
Overweight	50.82 (49.62–52.02)	24.21 (23.2–25.25)	14.5 (13.73–15.31)	10.47 (9.86–11.12)
Obese	38.38 (36.99–39.79)	24.61 (23.47–25.79)	17.82 (16.82–18.87)	19.19 (18.25–20.17)
Physical activity^*^				
No activity	36.64 (35.15–38.16)	24.51 (23.26–25.8)	17.61 (16.5–18.79)	21.24 (20.15–22.37)
Below recommendation	50.54 (49.09–51.98)	23.14 (21.99–24.33)	13.97 (13.08–14.91)	12.36 (11.53–13.24)
Above recommendation	56.47 (55.54–57.4)	23.17 (22.39–23.97)	12.31 (11.73–12.92)	8.04 (7.63–8.48)

^*^All the values are significant at p < 0.001.

### 3.4 Association between the sleep duration and chronic conditions

Table 4 presents the results of multinomial logistic regression showing the association between the number of existing chronic conditions (exposure) and sleep duration (outcome), using 7–9 h of sleep as the reference category. Both in their crude form and after adjusting for sociodemographic, socioeconomic position, and behavioral factors.

**Table 4:** Association between duration of sleep and chronic conditions among adult Canadians: CCHS 2017–18, Canada.

Chronic conditions	Less than 7 h		More than 9 h	
	OR (95% CI)	AOR (95% CI)	OR (95% CI)	AOR (95% CI)
No chronic condition (reference)	1.00	1.00	1.00	1.00
One chronic condition	1.14 (1.06–1.26)^**^	1.19 (1.09–1.30)^**^	1.67 (1.45–1.93)^**^	1.13 (0.92–1.39)
Two chronic condition	1.32 (1.21–1.44)^**^	1.38 (1.25–1.54)^**^	2.45 (2.09–2.88)^**^	1.37 (1.07–1.77)^*^
Three or more chronic conditions	1.54 (1.42–1.68)^**^	1.70 (1.52–1.91)^**^	4.81 (4.17–5.56)^**^	2.58 (1.96–3.41)^**^

Crude (unadjusted odds ratio (OR) with 95% Taylor Linearization) and adjusted (adjusted odds ratio (AOR) with 95% Taylor Linearization) odds ratios.

^*^ < 0.05, ^**^ < 0.001. Adjusted for sociodemographic, socioeconomic, and behavioral factors.

In the crude model, participants with three or more chronic conditions had significantly higher odds of reporting short sleep duration compared to those without chronic conditions (OR = 1.54;95% CI: 1.42–1.68, *p* < 0.001). After adjusting for sociodemographic, socioeconomic, and behavioral factors, this association remained statistically significant and slightly strengthened (AOR = 1.70; 95% CI: 1.52–1.91, *p* < 0.001).

**Figure 1:**
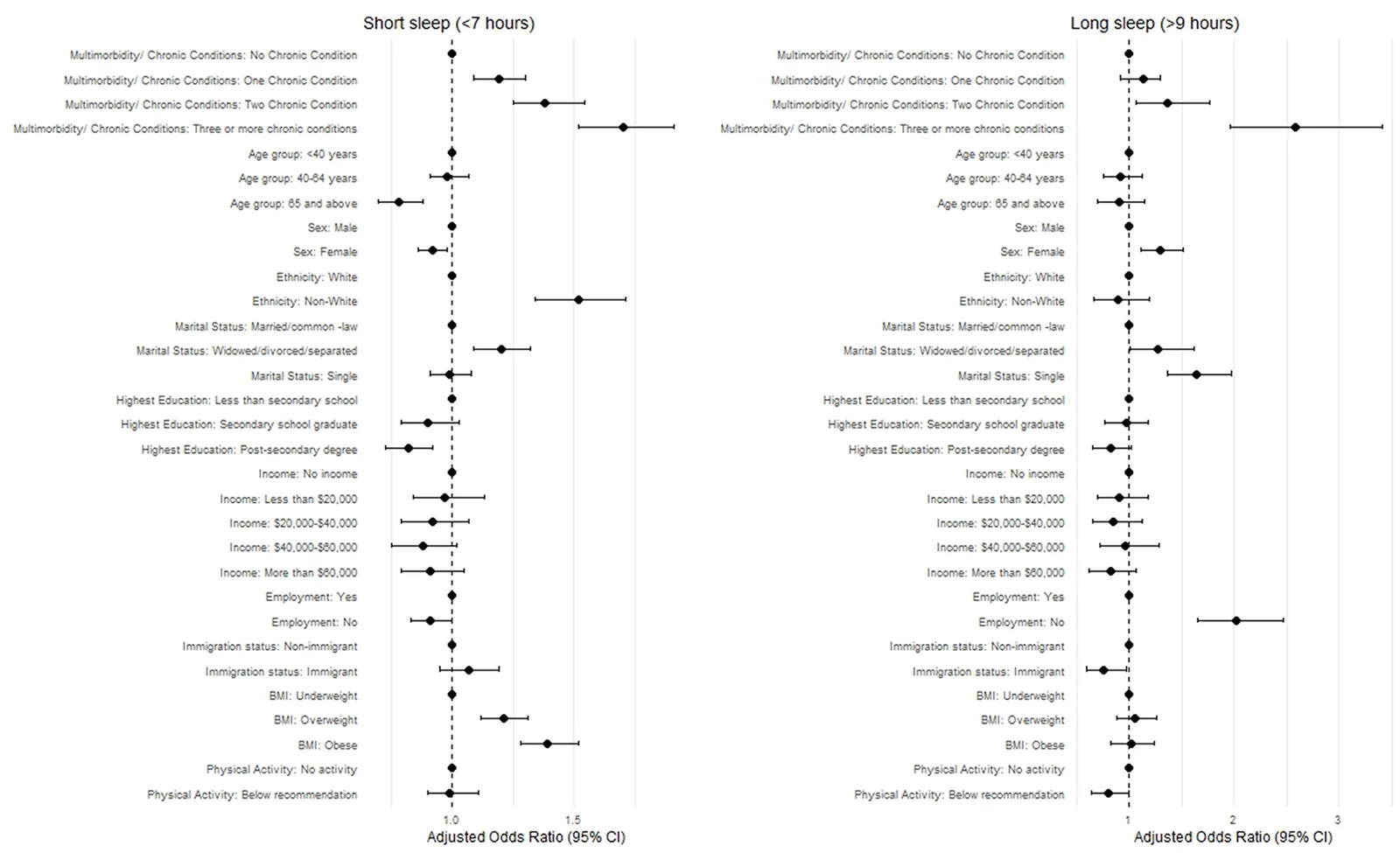
Adjusted (adjusted odds ratio (AOR) with 95% Taylor Linearization) association between duration of sleep and chronic conditions among adult Canadians: CCHS 2017–18, Canada.

A similar pattern was observed for long sleep duration. In the unadjusted analysis, individuals with three or more chronic conditions had substantially higher odds of long sleep compared to those without chronic conditions. After adjustment for potential confounders, participants with ≥3 chronic conditions had 2.58 times higher odds of long sleep (95% CI: 1.96–3.41; *p* < 0.001). Furthermore, Table 4 and Figure 1 show a graded increase in the odds of both short and long sleep duration with an increasing number of chronic conditions. This graded association suggests that as multimorbidity increases, the likelihood of reporting non-recommended sleep duration (either short or long) also increases, independent of sociodemographic, socioeconomic, and behavioral factors. The *p*-value for the trend was statistically significant (< 0.001).

## 4 Discussion

Results from this cross-sectional study show a high prevalence of short sleep duration and its association with chronic conditions among the study population.

In this study, the prevalence of short sleep duration was 40.96%. However, this is much higher than in other high-ranking socio-economic countries, such as Luxembourg, where only 5% reported sleeping less than 6 h (Ruiz-Castell et al., 2019). Adams et al. also found a low prevalence of shorter sleep hours among the Australian population (Adams et al., 2017). Variations in outcome measurements (e.g., defining sleep duration as less than 6 for Europeans and 5.5 h for Australians, instead of less than 7 h in our study) and differences in sample selection criteria (the European study considered only participants aged below 65 years) may account for these incongruent findings. These reflect the role of structural and behavioral factors on sleep health, which include increasing work-related stress, longer commuting time, extensive screen exposure, and the growing prevalence of shift work (Wickwire et al., 2017; Statistics Canada, 2023).

Importantly, our use of a < 7-h threshold aligns with current public health recommendations and may better capture subclinical sleep insufficiency that is nonetheless biologically meaningful (Hirshkowitz et al., 2015). Short sleep of this magnitude has been associated with dysregulation of inflammatory pathways, impaired glucose metabolism, and autonomic imbalance, which may contribute to the development and exacerbation of chronic diseases (Maloney and Kanaley, 2024; Direksunthorn, 2025). Arguably, our study used the same 9-h definition of long sleep duration as the Australian study, demonstrating close similarities in the prevalence, 7.73% for our study population and 8% for the Australian population (Adams et al., 2017). Krueger and Friedman (2009) reported a comparable prevalence for both short sleep duration (< 7 h) and long sleep duration (>9 h). Similar to our findings, they found that nearly half of the study population slept ≤ 7 h, and 8.5% reported sleeping more than 9 h (Krueger and Friedman, 2009). The similarity in long sleep prevalence between our study and the other studies suggests that long sleep duration may be relatively consistent across populations, although further cross-cultural studies are needed to determine whether this finding is generalizable. Moreover, the current study found that 11.65% of the study population was suffering from three or more chronic conditions, which is similar to previous research (Basham and Karim, 2019). Our study also found that one-fourth of the respondents had at least two chronic conditions, a proportion similar to that reported among both Canadian and American adults (Kader et al., 2024; Boersma et al., 2020). However, it is evident that the number of individuals with chronic conditions in the Canadian population is increasing annually. A 2015 study demonstrates that Canadians had only a 3.9% prevalence of more than three chronic conditions (Roberts et al., 2015). In the same population, the prevalence of chronic conditions increased three-fold from 2012 to 2018. The sharp increase in prevalence over time likely reflects a combination of population aging, improved survival from chronic disease, enhanced detection, and evolving disease classification practices (Public Health Agency of Canada, 2020). Additionally, lifestyle changes, including less physical activities and a sedentary lifestyle may further accelerate the accumulation of chronic conditions (Battista et al., 2025). On the other hand, Queenan et al. (2021) argued that methodological differences could explain the increased prevalence of chronic conditions in recent surveys. They found a higher prevalence (37%) of two or more chronic conditions among Canadian populations than in ours; however, they included different diseases than we did.

A key finding of this study is that individuals with three or more chronic conditions had significantly higher odds of reporting both short and long sleep duration compared with those without chronic conditions, independent of sociodemographic, socioeconomic, and behavioral factors. Our study results are in line with previously published articles (Ruiz-Castell et al., 2019; Beaudin et al., 2025; Chen et al., 2020; Lu et al., 2020). Like Nutakor et al. (2020), this research also indicates that individuals with three or more chronic conditions were prone to sleeping for longer hours. However, in our bivariate analysis, we found higher odds (4.81) for longer sleep. After adjusting for sociodemographic, socioeconomic, and behavioral factors, respondents with three or more chronic conditions reported approximately twice the odds of both short (AOR = 1.70) and long sleep (AOR = 2.58). This skewed result might be responsible for the effect of confounders in the long sleep group, which are still high compared to Peruvians (AOR = 1.29 for short and AOR = 1.45 for and long sleep duration; Mendez-Flores et al., 2023). Different cutoffs for sleep duration and chronic conditions in Peruvian semi-urban areas can justify the difference between their findings and ours.

However, not only did the study population report higher odds for long sleep duration, but it also showed an increasing pattern of chronic conditions in both short and long sleep durations. The result from the Luxembourg study shows a similar trend of increasing odds for a short sleep duration with increasing chronic conditions like ours (Ruiz-Castell et al., 2019). However, it did not exhibit a similar pattern in terms of long sleep duration. Their selected population group might explain this opposite directional result. The Luxembourg study enrolled respondents below 65 years; in contrast, our study found that long sleep is more prevalent among respondents aged 65 years and above, which aligns with the findings of Nutakor et al. (2020). This finding is also comparable with that of Itani et al. (2017) and Jike et al. (2018), which stated the importance of considering older age groups. Itani et al. (2017) found that short sleep was significantly associated with chronic conditions among participants aged below 65 years. On the other hand, Jike et al. (2018) identified long sleep duration as related to coronary heart disease among adults above 65 years. In older adults, long sleep may reflect underlying cardiovascular disease, frailty, or neurodegenerative processes, emphasizing the importance of age-stratified analyses when examining sleep–health relationships. Besides significant differences across the age group, the prevalence of long sleep duration significantly differed in other sociodemographic factors (i.e., marital status and education level) and socioeconomic status (employment status and household income). This suggests a potential for developing loneliness and social isolation, leading to increased sleeping duration (McLay et al., 2021).

This study possesses numerous strengths. This study covered a wide range of populations from four provinces and two territories. The findings from this study might be generalized to those provinces and territories. A wide range of chronic conditions were examined, which can inform healthcare providers in targeting, monitoring, and evaluating health outcomes. Furthermore, the findings highlight the association between sleep duration and chronic conditions, which may be valuable for policymakers in redesigning and implementing health programs for chronic conditions at a national level.

However, the study also has certain limitations. Due to its cross-sectional design, causal and temporal relationships cannot be established, the direction of association should be interpreted with caution. Sleep-related data were collected from a subsample, which excluded many individuals, particularly those in larger metropolitan areas and territories, during the sampling process. Although the CCHS is designed to be nationally representative, our analysis used a subsample with available sleep duration data, comprising approximately 50% of the original survey respondents. While survey weights were applied to account for the complex sampling design and non-response, the exclusion of participants without sleep data may have introduced selection bias and underrepresentation of certain population groups. Therefore, the results should be interpreted with caution regarding generalizability. Additionally, the data were self-reported, which may affect the accuracy of the measured indicators. Self-reported sleep duration from a single question may reflect time in bed rather than true sleep and does not capture insomnia, sleep quality, napping, or sleep regularity. Another limitation of this study is that the number of chronic conditions was measured by simple count, which assumes equal contribution of all conditions despite differences in frequency, clinical severity, and health impact. Future studies using weighted chronic conditions may better capture variations in disease burden and complexity. Consistent with other published studies, obesity was not included among the chronic conditions. Therefore, the specific contribution of obesity to sleep duration could not be directly evaluated. This is particularly relevant given the well-established association between obesity and sleep disturbances, including obstructive sleep apnea, which may influence both sleep duration and sleep quality. Although obesity (BMI) was included as a confounder and adjusted for in the final models, excluding it from the multimorbidity definition may have led to an underestimation of the overall burden of chronic conditions associated with abnormal sleep duration. Despite these limitations, our findings add to the evidence linking the number of chronic conditions with both short and long sleep duration among Canadian adults from selected provinces and territories. These findings underscore the importance of incorporating sleep duration into epidemiological research and public health surveillance of chronic conditions. Future longitudinal research is recommended to clarify the temporal and potentially bidirectional relationship between sleep duration and multiple chronic conditions. Future studies should also include broader geographic representation across all Canadian provinces and territories to improve generalizability. Additionally, examining vulnerable populations (e.g., older adults, immigrants, racialized groups, and individuals with obesity) separately may help identify population groups with a higher burden of multiple chronic conditions and abnormal sleep duration.

## Data Availability

Publicly available datasets were analyzed in this study. This data can be found here: https://www150.statcan.gc.ca/n1/en/catalogue/82M0013X2020001.
