# Supplementary material for: Association between sleep duration and co-existing chronic conditions in adults: insights from the 2017–2018 Canadian Community Health Survey
Source: Front Sleep. 2026 Sep 9;5:1714511. doi: 10.3389/frsle.2026.1714511 (PMC13597343; doi:10.3389/frsle.2026.1714511)
Supplement: Supplementary file 1 [file Table_1.docx]

**Supplementary table 1: Distribution of participants’ characteristics (in terms of weighted percentages) by sleeping hours: CCHS 2017-18, Canada**

|  | **In between 7-9 hours % (95% CI)** | **Less than 7 hours % (95% CI)** | **More than 9 hours % (95% CI)** |
| --- | --- | --- | --- |
| **Age group** | | | |
| <40 years | 53.13 (51.91-54.36) | 41.07 (39.87-42.29) | 5.79 (5.25-6.38) |
| 40-64 years | 50.98 (49.87-52.09) | 43.66 (42.57-44.76) | 5.36 (4.84-5.93) |
| 65 and above | 48.7 (47.53-49.88) | 35.32 (34.21-36.45) | 15.97 (15.03-16.96) |
| **Sex** | | | |
| Male | 50.56 (49.56-51.57) | 42.29 (41.3-43.29) | 7.14 (6.67-7.65) |
| Female | 52.04 (51.08-53.01) | 39.66 (38.72-40.6) | 8.30 (7.75-8.88) |
| **Ethnicity** | | | |
| White | 53.17 (52.41-53.92) | 38.93 (38.19-39.67) | 7.90 (7.51-8.32) |
| Non-White | 45.28 (43.42-47.14) | 47.90 (46.03-49.77) | 6.83 (5.88-7.91) |
| **Immigration status** | | | |
| Non-immigrant | 52.53 (51.77-53.3) | 39.71 (38.96-40.46) | 7.76 (7.36-8.17) |
| Immigrant | 47.64 (46.03-49.25) | 44.88 (43.28-46.49) | 7.48 (6.65-8.42) |
| **Marital Status** | | | |
| Married/common law | 52.55 (51.65-53.44) | 40.71 (39.83-41.59) | 6.74 (6.31-7.21) |
| Windowed/divorced/separated | 46 (44.53-47.49) | 42.99 (41.52-44.46) | 11.01 (10.02-12.09) |
| Single | 50.93 (49.43-52.42) | 40.54 (39.07-42.01) | 8.54 (7.72-9.43) |
| **Highest Education** | | | |
| Less than secondary school | 42.15 (40.37-43.96) | 41.34 (39.53-43.18) | 16.51 (15.13-17.98) |
| Secondary school graduate | 49.56 (48.05-51.06) | 42.12 (40.64-43.61) | 8.33 (7.52-9.21) |
| Post-secondary degree | 53.75 (52.88-54.62) | 40.64 (39.78-41.5) | 5.61 (5.22-6.03) |
| **Income** | | | |
| No income | 46.55 (44.15-48.96) | 43.25 (40.9-45.62) | 10.21 (8.97-11.59) |
| Less than $20,000 | 48.17 (46.53-49.83) | 41.46 (39.86-43.07) | 10.37 (9.44-11.38) |
| $20,000-$40,000 | 50.24 (48.49-51.98) | 40.27 (38.58-41.99) | 9.49 (8.51-10.58) |
| $40,000-$60,000 | 52.28 (50.42-54.13) | 39.73 (37.94-41.56) | 7.99 (7.05-9.04) |
| More than $60, 000 | 52.82 (51.79-53.84) | 41.02 (40.01-42.04) | 6.16 (5.66-6.71) |
| **Employment (Working status-last 12 month)** | | | |
| Yes | 52.83 (51.96-53.69) | 42.81 (41.95-43.67) | 4.36 (4-4.76) |
| No | 48.69 (47.33-50.07) | 38.06 (36.72-39.42) | 13.24 (12.27-14.28) |
| **BMI** | | | |
| Underweight | 56.56 (55.42-57.69) | 38.27 (37.17-39.39) | 5.17 (4.7-5.69) |
| Overweight | 51.98 (50.77-53.18) | 43.03 (41.82-44.23) | 5 (4.54-5.5) |
| Obese | 47.81 (46.43-49.19) | 42.17 (41.47-42.88) | 5.35 (4.74-6.02) |
| **Physical Activity** | | | |
| No activity | 47.3 (45.8-48.81) | 38.6 (37.16-40.07) | 14.09 (13.06-15.2) |
| Below recommendation | 52.5 (51.05-53.94) | 40.4 (38.99-41.83) | 7.10 (6.38-7.91) |
| Above recommendation | 52.41 (51.46-53.35) | 42.07 (41.14-43.01) | 5.52 (5.09-5.98) |

Footnote: all values are significant at p<0.001

**Supplementary table 2: Distribution of participants’ age (in terms of weighted percentages): CCHS 2017-18, Canada**

| **Age distribution across the study sample** | **Weighted % (95% CI)** |
| --- | --- |
| 18-19 years | 2.79 (2.53-3.08) |
| 20-24 years | 7.35 (6.94-7.77) |
| 25-29 years | 8.58 (8.16-9.01) |
| 30-34 years | 9.36 (8.97-9.77) |
| 35-39 years | 9.18 (8.78-9.61) |
| 40-44years | 8.10 (7.72-8.50) |
| 45-49 years | 7.95 (7.56-8.36) |
| 50-54 years | 8.40 (7.98-8.83) |
| 55-59 years | 8.96 (8.57-9.37) |
| 60-64 years | 8.55 (8.19-8.93) |
| 65-69 years | 7.15 (6.87-7.44) |
| 70-74 years | 5.67 (5.42-5.94) |
| 75-79 years | 3.79 (3.59-4.00) |
| Above 80 years | 4.15 (3.95-4.36) |
